# Taliaferro and Bozeman Controversy

**Published:** 1887-04

**Authors:** V. H. Taliaferro


### TALIAFERRO AND BOZEMAN CONTROVERSY.

### BOZEMAN'S UTERINE FORCEPS—THE "BUTTON-HOLE"—WHAT IT HAS DONE FOR THE PROFESSION AND FOR NATHAN BOZEMAN.

*To the Editors of the Atlanta Medical and Surgical Journal:*

GENTLEMEN—In the March issue of your journal there appears a communication from Dr. Bozeman, of New York, which purports to be a reply to my paper upon the "Intra-uterine Tampon," published in the February issue of the same journal. By his complete silence upon the issues on which he was arraigned at the professional bar he makes a virtual acknowledgment of guilt. It is true he does make a sickly effort to show by letters from abroad that his "button-hole" was thought well of *thirty years ago*, but his showing is too puerile for respectful notice. If, indeed, his letters prove anything it is that he is most thought of where least known.

While making no reply to my *exposé* of the dishonorable and unprofessional methods of hoisting himself to professional notoriety and publicity, he has the unblushing effrontery to hide himself behind a labored and exhaustive effort to prove that I appropriated *his uterine dressing forceps.* I fear the poor man is crazy, and merits charity instead of rebuke. It does, indeed, look too silly and preposterous that I should have to deny ownership in a thing I never made the least vestige of claim to, either in writing, by word or by act. As stated in my paper, any small-bladed uterine dressing forceps answers for the *intra-uterine tampon*. In many of the varieties of dressing forceps there is the merest shade of difference in shape, and in office work we do not confine ourselves to any one variety. Among other dressing forceps on our table is a modified Bozeman, but nowhere or at any time did we ever make claim of the instrument or its modification, and its use in the illustrations was more the result of accident than preference. My associate, Dr. Noble, makes the following written statement:

"For the past four or five years I have ordered all instruments used by our firm (Taliaferro & Noble), and when Bozeman's instrument, or any modification of it, was wanted I always specified 'Bozeman's forceps,' etc. When we have presented these instruments (forceps) to our friends or at the clinics of the Atlanta Medical College we have always given Bozeman credit for them making no claim even for our modification.

Respectfully, GEO. H. NOBLE.

To DR. V. H. TALIAFERRO."

Mr. Isaac Phillips, of this city, through whom we make most of our orders, makes me the following written statement:

"In ordering from the manufacturers Bozeman's uterine dressing forceps for yourself or others, I have invariably designated them as such. In selling the instrument to doctors and students, I have always given them the name of Bozeman. Knowing that you and Dr. Noble had made an important modification of the Bozeman instrument, I applied to you for the privilege of publishing a cut of it bearing your name, which you refused, stating that the instrument was Bozeman's.

Very truly yours,

ISAAC PHILLIPS.

To V. H. TALIAFERRO, M. D."

Do we need further evidence that the man has lost his head? Let us see: He says I have engaged in this controversy merely to advertise myself by associating my name with great men. *He regards himself a great man!* Phew! *what a hallucination.* As a literary kleptomaniac he is without a rival; his *greatness*

beyond this is unknown, save by his own statement. Dr. Bozeman's professional literature for the past thirty years, in volubility and space, is immense, but emasculated of the self-appropriated work of others and simmered down to bottom facts, the "button-hole suture" stands alone as the prominent figure-head to mark the man's professional status, or rather his want of it. The only use I know of the profession making of the "button-hole suture" is to let it alone.

The New York Academy of Medicine twice put this professional crank under the ban, first by sustaining and publishing the scathing denunciation of Bozeman by Sims; second, by its sharp rebuke of the disgraceful effort to outlaw Fordyce Barker from the Academy. Professional contempt finally culminated at a late meeting of the American Gynecological Association, when it was determined to stand the old song of the "button-hole" no longer, and with the foot of silent indignation this professional fungus was hurled from its midst. Very truly yours,

V. H. TALIAFERRO.

